# Non-Invasive Continuous Blood-Pressure Monitoring Models Based on Photoplethysmography and Electrocardiography

**DOI:** 10.3390/s19245543

**Published:** 2019-12-15

**Authors:** Haiyan Wu, Zhong Ji, Mengze Li

**Affiliations:** 1 College of Bioengineering, Chongqing University, Chongqing 400044, China; 201719021053@cqu.edu.cn (H.W.); 20136471@cqu.edu.cn (M.L.); 2 Chongqing Medical Electronics Engineering Technology Center, Chongqing 400044, China

**Keywords:** characteristics of pulse waveform, error back-propagation neural network, model integration, multiple population genetic algorithm, noninvasive continuous blood pressure monitoring

## Abstract

Blood pressure is an extremely important blood hemodynamic parameter. The pulse wave contains abundant blood-pressure information, and the convenience and non-invasivity of its measurement make it ideal for non-invasive continuous monitoring of blood pressure. Based on combined photoplethysmography and electrocardiogram signals, this study aimed to extract the waveform information, introduce individual characteristics, and construct systolic and diastolic blood-pressure (SBP and DBP) estimation models using the back-propagation error (BP) neural network. During the model construction process, the mean impact value method was employed to investigate the impact of each feature on the model output and reduce feature redundancy. Moreover, the multiple population genetic algorithm was applied to optimize the BP neural network and determine the initial weights and threshold of the network. Finally, the models were integrated for further optimization to generate the final individualized continuous blood-pressure monitoring models. The results showed that the predicted values of the model in this study correlated significantly with the measured values of the electronic sphygmomanometer. The estimation errors of the model met the Association for the Advancement of Medical Instrumentation (AAMI) criteria (the SBP error was 2.5909 ± 3.4148 mmHg, and the DBP error was 2.6890 ± 3.3117 mmHg) and the Grade A British Hypertension Society criteria.

## 1. Introduction

Blood pressure is the combined result of ventricular ejection and peripheral resistance. Blood pressure is divided into arterial blood pressure and venous blood pressure, and blood pressure without any specific descriptors generally refers to arterial blood pressure [1]. Hypertension is an important cause of cardiovascular and cerebrovascular diseases, and it is significantly correlated with strokes and coronary heart disease [2]. According to the World Health Statistics 2018 published by the World Health Organization, in 2016 the number of deaths caused by cardiovascular diseases was as high as 17.90 million globally, accounting for 44% of deaths due to non-communicable diseases [3].

An accurate and real-time blood-pressure value is profoundly important for the diagnosis, prevention, and treatment of hypertension-related diseases [4]. However, blood pressure fluctuates and is susceptible to physiological, psychological, and environmental factors. The intermittent blood-pressure measurements vary widely and cannot reflect an individual’s true blood pressure accurately, such as the “white-coat hypertension” and “masked hypertension” [5]. Real-time monitoring of blood pressure by heartbeat would provide a more adequate basis for clinical treatment [6,7]. The use of arterial catheterization to achieve a direct measurement of blood pressure per cardiac cycle is the gold standard for blood-pressure measurement. However, it is accompanied by serious complications, such as intermittent vascular occlusion and local infection. In addition, the equipment is expensive and complicated to operate [8]. To overcome the shortcomings of invasive blood-pressure measurement techniques, the non-invasive continuous blood-pressure measuring techniques emerged at the right time. Currently, the non-invasive continuous blood-pressure measurement methods mainly include the arterial tonometry method, vascular unloading technique, pulse wave velocity (PWV) method, and pulse waveform characteristic parameter method.

The arterial tonometry method is applicable to superficial arteries such as the radial artery [9]. Calibration is not needed every time, and long-term blood-pressure measurement can be achieved; nevertheless, the sensor is highly sensitive to displacement and pressure [10]. The vascular unloading technique accomplishes blood-pressure monitoring using the mechanical properties of the blood vessel [11]. However, long-term measurement may cause venous congestion and affect measurement accuracy. The theoretical basis of the PWV method is the Moens–Korteweg equation. Gribbin varied the arterial dilatation pressure of the limb in a large range by exerting external pressure, monitored the PWV, and found that PWV was closely related to blood pressure [12]. In 1984, Tanaka calculated the blood-pressure values based on PWVs for the first time; however, the predicted values had big standard errors [13]. Similar to the theory of PWV, Payne et al. verified that the pulse wave transit time (PWTT) was associated with blood pressure [14], and the correlation between PWTT and systolic blood pressure (SBP) was stronger than that between PWTT and diastolic blood pressure (DBP) [15,16]. McCombie presented a framework using peripheral artery PWV and blind system identification to estimate blood pressure [17]. The PWV method solved the issues of complicated device and uncomfortability; still, this model had weak generalization ability due to individual differences [18].

In recent years, scholars have extracted the features of the pulse wave signals in the time and frequency domains and constructed multivariate regression models [19,20,21,22]. The structures of the regression models were similar to the PWTT-based models, while the features changed from univariate to multivariate and the accuracy improved. With the development of machine learning, the characteristic parameters of models were further enriched, including the amplitude, phase characteristics of pulse waves extracted with fast Fourier transform [23], spectral characteristics [24], and the features of the photoplethysmography (PPG) waveform and related first and second (time) derivatives [25,26]. Moreover, the model construction methods were expanded, such as neural network [24,27,28,29], support vector machine [30], adaptive boosting regression [31], and random forest algorithm [32]. The blood-pressure estimation methods based on machine learning and big data covered more blood-pressure information and improved the estimation accuracies of the models to some extent. However, numerous factors affect blood pressure. During modeling, finding an appropriate feature set can undoubtedly reduce the complexity of the blood-pressure prediction models and be more conducive to the real-time monitoring of blood pressure.

The present study extracted as many waveform characteristics related to blood pressure as possible based on PPG in combination with electrocardiography (ECG) signals, as well as an individual’s characteristic features. Furthermore, the neural network was used as the mainline, the impact of the extracted features on the model output was analyzed, and then an appropriate feature set was selected. Moreover, the blood-pressure estimation model was constructed based on the selected features and optimized with optimization algorithm. Finally, the estimation accuracies of the proposed models were explored according to the selected criteria and compared models.

## 2. Materials and Methods

### 2.1. Experimental Data Collection

The experiments were approved by the Ethics Committee of the First Affiliated Hospital of Third Military Medical University, and the date of approval was 4 November 2013. and all subjects gave their informed consent for inclusion before they participated in the study. The study was conducted in accordance with the Declaration of Helsinki. The hardware platform for experimental data acquisition included a non-invasive cardiac function analyzer prototype developed independently in this study and an Omron electronic sphygmomanometer (HEM-7210) (OMRON Corporation, Kyoto, Japan). The non-invasive cardiac function analyzer prototype was used to synchronously collect the fingertip photoplethysmogram signals and ECG signals of the individuals. A lead-II ECG configuration was used. The photoplethysmogram sensor was clamped onto the tip of the right index finger of the individual and kept at the same level with the heart, and the signal sampling frequency was set at 500 Hz. The Omron electronic sphygmomanometer was used to measure the blood pressure at the corresponding time. The cuff was worn on the left upper arm of the individual, and the electronic sphygmomanometer was used in strict accordance with the manuals [33].

A total of 27 healthy subjects (13 male and 14 female) aged 18–27 years were enrolled in this study. The recruitment of participants was based on voluntary and informed consent, and the participants had no conflicts of interest with research contents and results.

The static and dynamic experiments were performed at room temperature, and the dynamic experiment was a random-number mental arithmetic [34]. During the experiment, the PPG and ECG signals were continuously collected (it should be noted that the duration of each signal acquisition both static and dynamic experiments was within 20 min). After both signals’ waveforms had not changed much and were in a relatively stable state, the blood pressure was measured with an Omron electronic sphygmomanometer at 1 min intervals. After removing the useless waveforms, a total of 875 sets of data (583 sets of the data were static condition, and the rest were dynamic condition) were recorded in the experiment. Each set of data consisted of the height, weight, gender, and PPG and ECG signal waveforms with a duration of 20 s of the individuals, and the measured values of SBP and DBP at the corresponding time.

### 2.2. Signal Preprocessing and Characteristic Parameter Extraction

**Figure 1:**
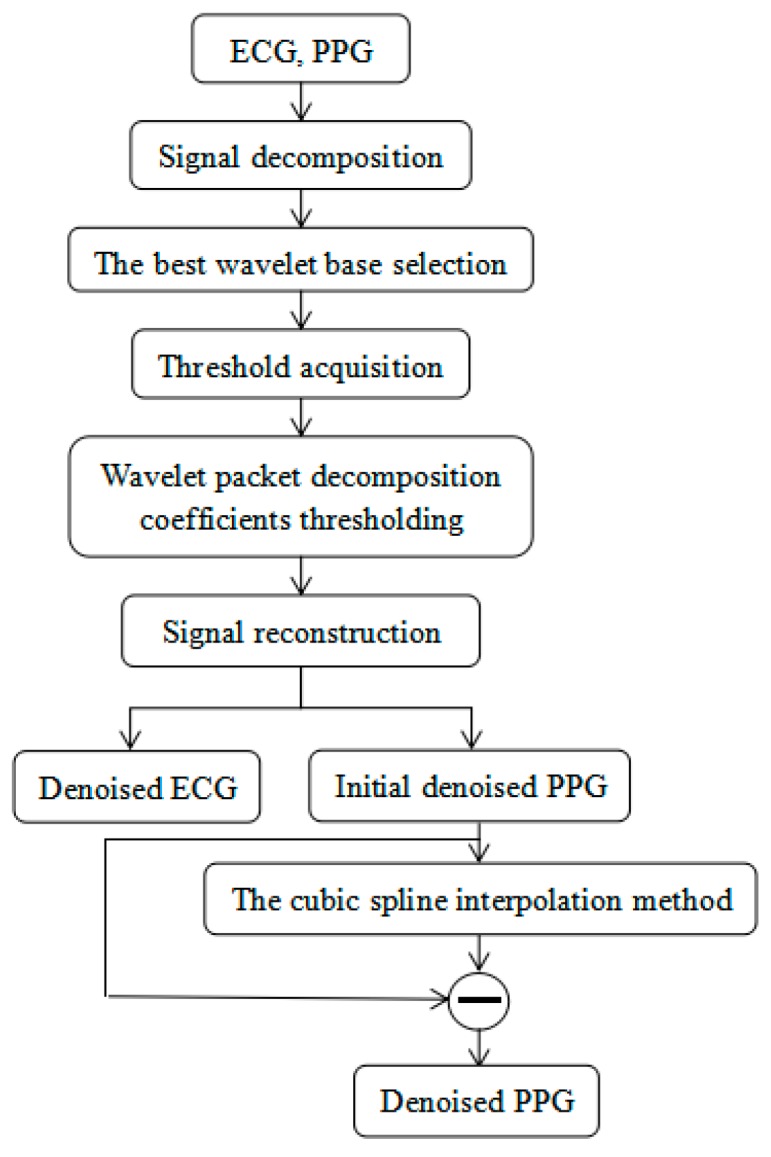
The algorithm flow chart of the signal preprocessing.

The ECG and PPG signals are weak physiological signals susceptible to industrial frequency, tremor, and breathing during signal collection. The denoising of ECG signals and PPG signals is indispensable to ensure accurate extraction of signal characteristics. In this study, the wavelet packet method was employed to denoise the ECG and PPG signals on the MATLAB 2016b platform (MathWorks, Natick, MA, USA) [35], and the PPG signals were further denoised by the cubic spline interpolation method. The algorithm flow chart of the signal preprocessing is shown in Figure 1.

**Figure 2:**
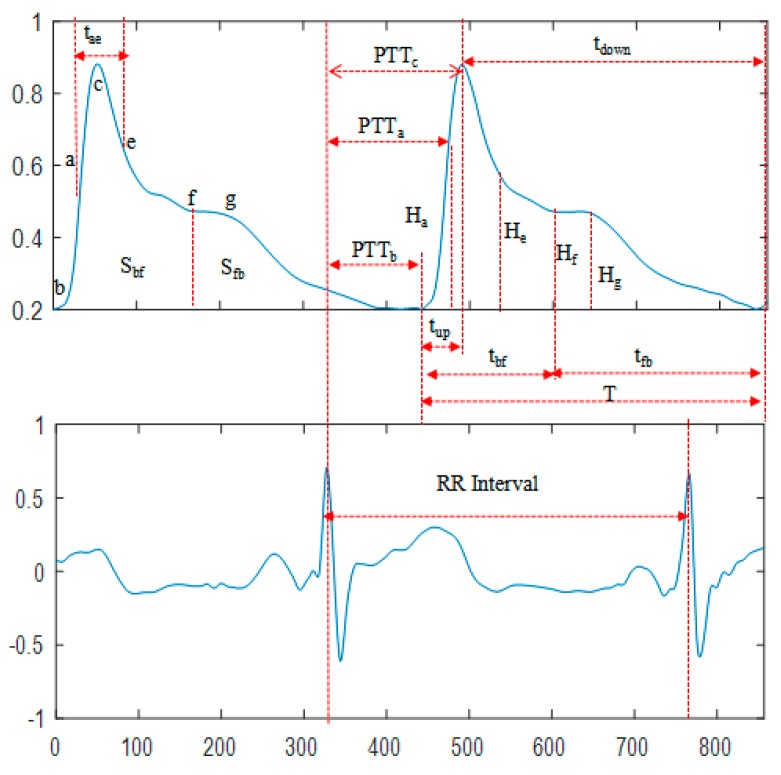
Schematic diagram of features.

The time parameters were extracted from the denoised ECG and PPG signals, including the time from the pulse starting point b to the peak point c (t_up_) and to the f point of the dicrotic notch (t_bf_); the time from c and f to the point b of the next cardiac cycle (t_down_ and t_fb_, respectively); the time from the point a with maximum ascending slope to the point e with minimum descending slope of the photoplethysmography (t_ae_); the ratio of the aforementioned time parameters to the cardiac cycle (t_upr_, t_bfr_, t_downr_, t_fbr_, and t_aer_); as well as the time from the R-wave of ECG to the photoplethysmography points b, a, and c within the same cycle (PTT_b_, PTT_a_, and PTT_c_, respectively). The amplitude parameters were the ratio of the amplitude of the points a, e, f, and g to the amplitude of the point c (H_ar_, H_er_, H_fr_, and H_gr_, respectively). The area parameters were the systolic area S_bf_ and the diastolic area S_fb_. Other types of parameters also existed, such as the K value (K = (S_m_−H_c_)/(H_c_−H_b_), S_m_ is the mean pulse wave area of the cycle), the heart rate (HR) calculated by the adjacent R-wave interval, and the main wave rising slope (C_slope_). A total of 22 waveform characteristics were included, and a schematic diagram of some features is shown in Figure 2. In addition, two characteristics of the individuals were introduced: Gender and body mass index (BMI) (there was no significant difference in age between participants, so age was not considered in individual characteristics).

### 2.3. Model Construction

#### 2.3.1. Back-Propagation (BP) Neural Network

The error back-propagation (BP) algorithm has a simple network structure, a powerful parallel processing capability, and a strong fault tolerance, and can approximate the complex non-linear relationships well [36]. The BP neural network is a multilayer feedforward neural network with three or more layers, including an input layer, a hidden layer or layers, and an output layer. The BP neural network algorithm includes the forward propagation, determining whether to go to the back-propagation, error back-propagation, and final result output. The forward propagation propagates the input vector from the input layer to the output layer via the hidden layer(s). The determination of whether to go to the back-propagation involves calculating whether the actual output of the input layer matches the expected output criteria and whether the number of train epochs reaches the preset number of epochs. The process of error back-transmission propagates the output error in some form to the input layer via the hidden layer, adjusts the rule according to the weights, and corrects the connection weights. The process of producing the final result output is conducted when the network output error reduces to the expected error or the network train epochs reach the preset epochs [37].

#### 2.3.2. Feature Parameter Screening

Excessive input parameters or redundancy can increase network complexity and even reduce accuracy in modeling. Therefore, the characteristic parameters mentioned in Section 2.2 were effectively screened to include the significant features and exclude the non-significant features in the blood-pressure models. This way, the model was simplified, the model accuracy was improved, and the model was more favorable for the continuous monitoring of blood pressure. In this study, the variable screening was performed using the BP neural network with the mean impact value (MIV) [38,39] method, finding out the characteristic parameters with big impacts on the model output. The detailed process was as follows:

1. The BP network was trained with the original input sample P.
2. Two new training samples P1 and P2 were obtained by adding and reducing 10% of the original values of one target characteristic variable while keeping the others constant.
3. P1 and P2 were used as the simulation samples to perform simulation by the network established with (1), and two simulation results A1 and A2 were produced.
4. The difference between A1 and A2 was calculated to obtain the impact value of this characteristic variable on the model output.
5. The MIV was calculated based on the existing sample size, that is, the MIV value of this characteristic variable.
6. Steps (2)–(5) were repeated to sequentially calculate the MIV of each characteristic variable in the sample P.
7. The weight of the impact of each characteristic variable was calculated on the prediction result using the following Equation (1):
  (1) $$\partial_{i}=\left|MIV_{i}\right|/\sum_{i=1}^{m}\left|MIV_{i}\right|$$
  where $\partial_{i}$ is the relative contribution of the i^th^ independent variable and m is the number of independent variables in the sample P. Moreover, $\sum_{i=1}^{n}\partial_{i}/\sum_{i=1}^{m}\partial_{j}$ is the cumulative contribution of the first n independent variables. For selecting the input variables of the BP neural network, the cumulative contribution needs to be greater than 85% [38].

#### 2.3.3. BP Neural Network Parameter Optimization

The BP neural network is the most widely applied algorithm among artificial neural networks. However, it has some disadvantages, such as slow learning-convergent velocity, difficulty in network structure determination, and inability to obtain accurate initial connection weights and thresholds. This study adopted the multiple population genetic algorithm (MPGA) to optimize the neural networks in an attempt to compensate for the aforementioned drawbacks of BP neural network [36].

The genetic algorithm (GA) is a random and self-adaptive search algorithm for global optimization developed by borrowing the ideas of natural selection and a random evolutionary mechanism [40]. It does not depend on the gradients and has strong robustness and global search capability; however, its premature convergence issue still cannot be ignored. The specific manifestation is that all individuals in the population converge to the same state and the evolution is stopped, reaching unsatisfactory results. However, MPGA was proposed targeting the premature convergence problem of GA, and mainly introduced the following three strategies [41]:

1. The single population genetic evolutionary framework of GA was broken, and multiple populations were introduced to perform optimization search simultaneously. The control parameters were different for each population.
2. Various populations were connected by immigration operators, and multiple populations co-evolved.
3. The artificial selection operators were used to save the best individuals of each population in each generation, and the best individual and the least preserved generation was regarded as the criterion for terminating the algorithm.

**Figure 3:**
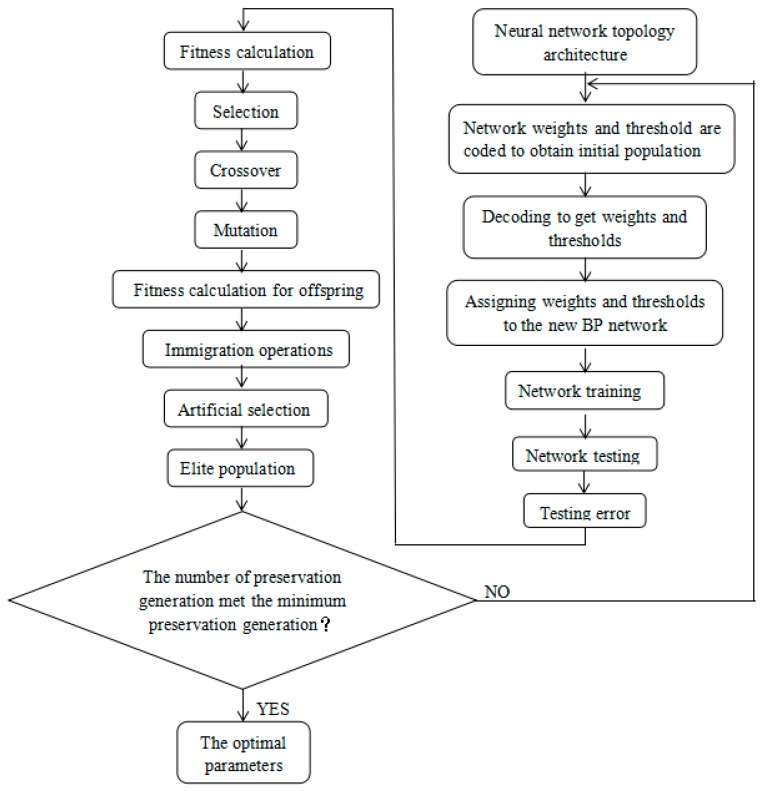
The algorithm flow chart of multiple population genetic algorithm (MPGA) in optimizing the back-propagation (BP) neural network.

The algorithm flow chart of MPGA in optimizing BP neural network is shown in Figure 3. The detailed steps were as follows:

1. Binary coding. Each weight and threshold were binary coded. The codes of all the weights and thresholds comprised the code for an individual; multiple individuals formed a population; and multiple populations were initialized in parallel.
2. Fitness calculation. For the multiple populations of (1), the fitness function was substituted to calculate the fitness value of each individual in each population.
3. Selection, crossover, and mutation. The selection operators were used for selection, and the crossover probability and mutation probability were used to generate offspring.
4. Fitness calculation for offspring.
5. Immigration operations. The best individuals in the population were identified to perform immigration operations.
6. Artificial selection. The best individual of each population was collected by artificial selection operator and put into an elite population for preservation.
7. The best individual in the elite population was found, and the number of preservation generations for this best individual was updated. When the number of preservation generations met the minimum preservation generation, the algorithm was terminated, and the optimal individual was decoded to generate the optimal parameters.

#### 2.3.4. Model Integration

A big variation exists between the blood pressure and the corresponding waveform characteristics of an individual. In the current circumstances, this study only considered two characteristics of individuals, BMI and gender, which were definitely not sufficient. Moreover, to reduce overfitting, this study trained multiple independent blood-pressure models and integrated the models using different proportional coefficient set for different individuals, achieving continuous monitoring of individualized blood pressure. In this study, MPGA was used to determine the proportional coefficients of these models, and finally to get the individualized blood-pressure monitoring models.

## 3. Results and Analysis

### 3.1. Summary of Data Information

The detailed information of the experimental data is shown in Table 1.

**Figure 4:**
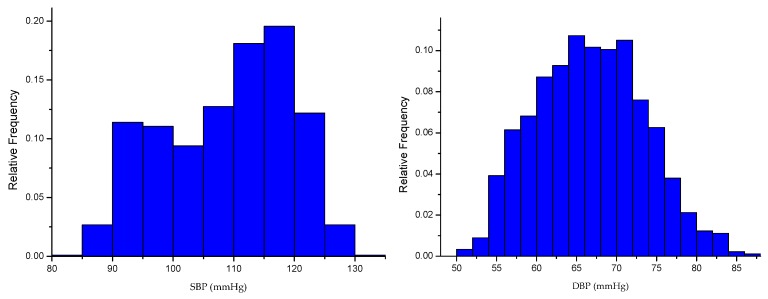
The distribution of blood pressure obtained from the experiment.

The distribution of blood pressure obtained from the experiment is shown in Figure 4.

### 3.2. Signal Preprocessing

**Figure 5:**
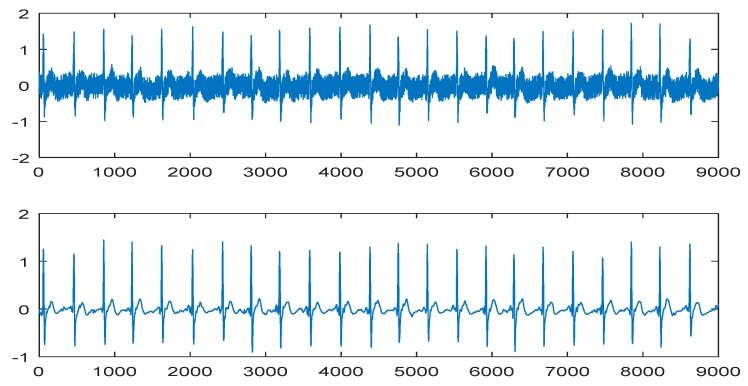
Comparison of the electrocardiography (ECG) signal before (top) and after (bottom) denoising.

Based on the nature of the wavelet basis and the ECG signals that need to be processed, “coif5” was used as the wavelet packet base. The number of decomposition layers was set to 3, and “SURE entropy” was adopted as the information cost function to select the best wavelet base. The “ddencmp” function was applied to acquire the denoising threshold according to the nature of the signal. Then, the wavelet coefficients corresponding to the optimal wavelet basis were quantized, and finally the denoised signals were obtained via the reconstruction algorithm. The denoising results are shown in Figure 5.

**Figure 6:**
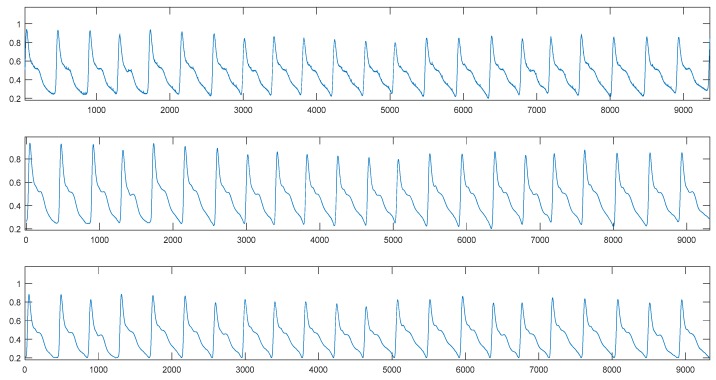
Comparison of the photoplethysmography (PPG) signal before (top), initial (middle) and after (bottom) denoising.

For the PPG signals, the initial denoising and processing were similar to the ECG signals. Nevertheless, for the baseline drift with great influence, this study treated it further using the cubic spline interpolation method [38]. The PPG denoising results are shown in Figure 6.

### 3.3. Models

The BP neural network used in this study was based on the default Neural Network Toolbox of MATLAB 2016b. The MPGA was based on the Sheffield Genetic Algorithm Toolbox. The data from 17 individuals, Test 1–Test 8 and Test 14–Test 22, a total of 775 sets of data, were disordered first and used for the basic blood-pressure model construction. The data from the other 10 individuals, Test 9–Test 13 and Test 23–Test 27, a total of 120 sets of data, were used for the individual’s blood-pressure model construction and testing.

#### 3.3.1. BP Models

A three-layer BP neural network can implement arbitrary non-linear mapping if the number of nodes in the hidden layer is not limited. This study selected a single hidden layer BP neural network to improve the training speed and simplify the neural network structure. It was applied to the 775 sets of data to construct basic models. Then, the 24 parameters, including the waveform characteristic parameters and the individual’s features, were used as input, and the corresponding SBP and DBP values measured by the sphygmomanometer were used as output to construct one BP blood-pressure model regarding SBP (nets0) and one BP blood-pressure model regarding DBP (netd0).

The BP neural network parameters were set as follows: The learning rate was 0.01, the activation function of the hidden layer was “tansig”, the activation function of the output layer was “purelin”, and the learning algorithm was the Levenberg–Marquardt algorithm with fast convergence, which was the “Trainlm”. With respect to the setting of the number of nodes in the hidden layer, the following empirical equation was used (Equation (2)):

(2) $$m=\sqrt{n+l}+\partial$$

where *m* is the number of nodes in the hidden layer, *n* is the number of nodes in the input layer, *l* is the number of nodes in the output layer, and ∂ is a constant between 1 and 10. The range of nodes of the hidden layer was [6,16]. As the establishment of the basic model was only for parameter screening, the value of *m* only needs to be set within the range. In this study, it was set to 11.

#### 3.3.2. BP with Mean Impact Value (MIV–BP) Models

Based on the BP neural network nets0 and netd0 constructed in Section 3.3.1, the impact of each input feature on the model output was analyzed by MIV. As a variety of characteristic parameters were involved in this study, two rounds of MIV–BP parameter screening were performed. The first round was a rough screening to select the input characteristics contributing to 91% of cumulative contribution ratio. The features included for SBP were BMI, gender, PTT_c_, PTT_a_, HR, t_bf_, t_fb_, t_ae_, t_down_, t_up_, t_bfr_, t_fbr_, t_upr_, t_downr_, t_aer_, H_er_, H_fr_, and K; and for DBP were BMI, PTT_c_, PTT_a_, HR, t_bf_, t_fb_, t_ae_, t_down_, t_up_, t_bfr_, t_fbr_, t_upr_, t_downr_, t_aer_, H_ar_, H_er_, H_gr_, and K. The second round was based on the results of the first round. The orders of the MIV values of the input features of the SBP and DBP models are shown in Table 2 and Table 3, respectively. The features contributing to 89% of cumulative contribution ratio were included. Therefore, 14 features were selected for SBP (including BMI, t_downr_, t_bf_, t_fbr_, PTT_c_, t_up_, t_bfr_, t_down_, H_er_, t_aer_, t_upr_, HR, K, and PTT_a_) and14 for DBP (including t_downr_, H_er_, t_bf_, t_fbr_, BMI, t_bfr_, t_ae_, t_up_, HR, t_aer_, t_fb_, t_down_, t_upr_, and PTT_a_).

#### 3.3.3. Multiple Population Genetic Algorithm (MPGA)–MIV–BP Models

In addition to the input characteristics identified in Section 3.3.2, the number of nodes of the hidden layer needed to be determined in order to find out the topology of the BP neural models. According to Equation (2), the node range of the hidden layer of the basic BP models for SBP and DBP was 5–14 after feature screening. In this study, the 775 sets of data used for the basic model construction were subjected to fivefold cross-validation (5-CV) to calculate the root mean squared error (RMSE) (Equation (3)) of the network, while holding the other parameter settings consistent as in Section 3.3.1. The optimal number of nodes within the node range of the hidden layer was then determined (Equation (3)).

(3) $$RMSE=\sqrt{\left[\sum_{i=1}^{n}\left(y_{i}-{\hat{y}}_{i}\right)\right]/n}$$

where $y_{i}$ is the measured blood pressure, ${\hat{y}}_{i}$ is the predicted blood pressure, and n is the sample size.

The RMSE values for SBP and DBP of the BP neural network for various nodes of the hidden layer are shown in Table 4. It was determined that the number of nodes in the hidden layer was 12 for SBP and 10 for DBP in the BP neural network.

**Figure 7:**
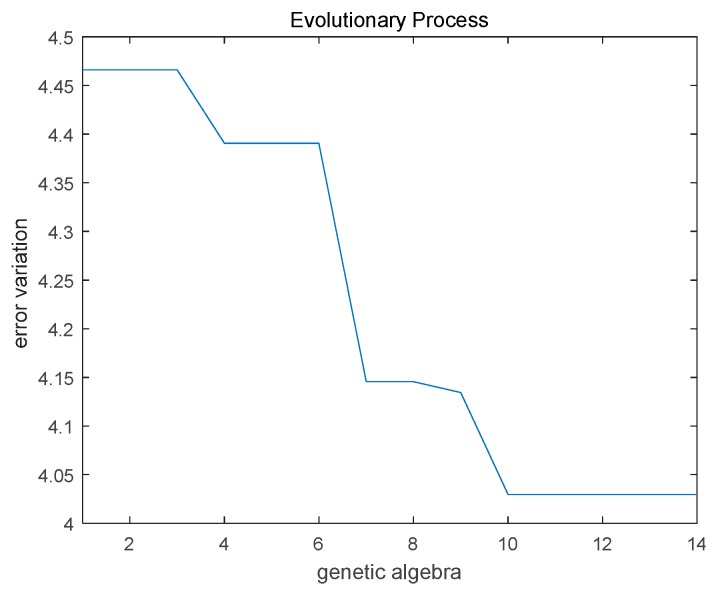
The evolution curve of Nets1.

The parameter setting of MPGA in optimizing the BP neural network was as follows: The range for weights and threshold of the network was −0.5–0.8; the individual number in an initial population was 20, the binary digit for an individual was 10, the population number was 5, the preservation generation for the best individual was 3, the crossover probability was randomly generated within the range of 0.7–0.9 the mutation probability was randomly generated within the range of 0.001–0.05 and the output of the target function was network RMSE. Based on the 775 sets of data for the basic model construction, 5-CV was applied to train 5 independent SBP networks (Nets1, Nets2, Nets3, Nets4, and Nets5) and 5 independent DBP networks (Netd1, Netd2, Netd3, Netd4, and Netd5). The evolution curve of Nets1 is shown in Figure 7.

For the same data samples (training set and test set), the RMSE values before and after optimization are shown in Table 5. By comparison, it was found that the prediction of BP neural network improved significantly after MPGA optimization.

#### 3.3.4. Integrated Models

The continuous blood-pressure monitoring models for an individual could be obtained using the networks trained as in Section 3.3.3 (Nets1, Nets2, Nets3, Nets4, and Nets5 for SBP and Netd1, Netd2, Netd3, Netd4, and Netd5 for DBP) to construct SBP and DBP prediction models for individuals, as shown in the following equations (Equations (4) and (5)):

(4) $$NETs=a_{1}*Nets1+a_{2}*Nets2+a_{3}*Nets3+a_{4}*Nets4+a_{5}*Nets5$$

(5) $$NETd=b_{1}*Netd1+b_{2}*Netd2+b_{3}*Netd3+b_{4}*Netd4+b_{5}*Netd5$$

The MPGA was used to determine the proportional coefficients $a_{1}$, $a_{2}$, $a_{3}$, $a_{4}$ and $a_{5}$ of the SBP prediction model of an individual and the coefficients $b_{1}$, $b_{2}$, $b_{3}$, $b_{4}$ and $b_{5}$ of the DBP prediction model of an individual. The optimization parameters of MPGA were set as follows: The range for proportional coefficients was −0.5−0.8 the individual number in an initial population was 20, the binary digit for individual was 25, the population number was 15, the preservation generation for the best individual was 3, the crossover probability was randomly generated within the range of 0.7–0.9 the mutation probability was randomly generated within the range of 0.001–0.05 and the output of the target function was network RMSE. The data of the Test 9–Test 13 and Test 23–Test 27, a total of 120 sets of data, were used for the construction and testing of the blood-pressure models of individuals. For each individual, the first six sets of data were used to determine the proportional coefficients of the individual’s continuous blood-pressure monitoring models, and the remaining six sets were used as the test set for the individual’s monitoring model. The proportional coefficients of the SBP and DBP prediction models of different individuals and the network RMSE values during optimization are shown in Table 6 and Table 7.

### 3.4. Predicted Results and Comparison

The RMSE, mean absolute deviation (MAD) (Equation (6)), and standard deviation (STD) (Equation (7)) were used to evaluate the model prediction capability. For the individualized models from Section 3.3.4, the remaining samples, which were the last six sets of data of Test 9–Test 13 and Test 23–Test 27, were used to validate the blood-pressure models (Equations (6) and (7)).

(6) $$MAD=\left(\sum_{i=1}^{n}\left|y_{i}-{\hat{y}}_{i}\right|\right)/n$$

(7) $$STD=\sqrt{\left(\sum_{i=1}^{n}{\left(y_{i}-{\hat{y}}_{i}-\bar{y_{i}-{\hat{y}}_{i}}\right)}^{2}\right)/\left(n-1\right)}$$

where $y_{i}$ is the measured blood pressure, ${\hat{y}}_{i}$ is the predicted blood pressure, and n is the sample size. The model prediction results are shown in Table 8.

**Figure 8:**
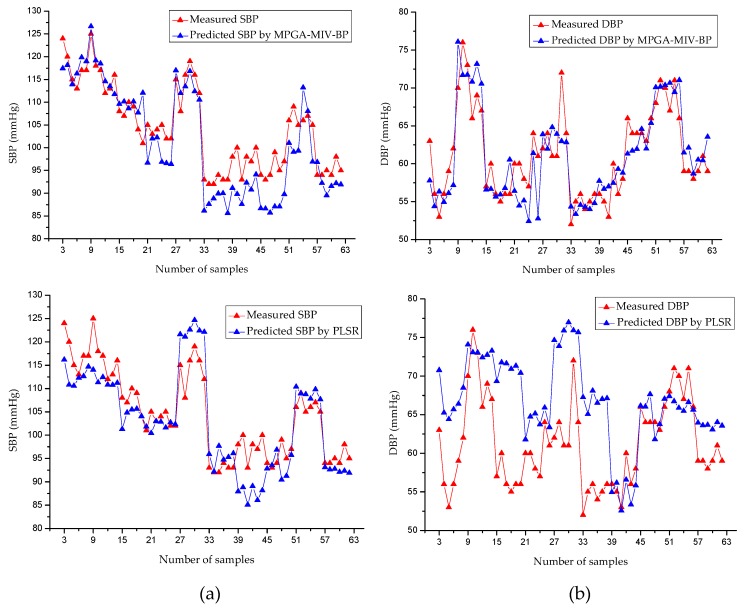
Comparison of predicted values and actual values. (**a**) SBP models. (**b**) DBP models. For both (**a**,**b**), from top to bottom, MPGA-MIV-BP, partial least squares regression (PLSR) models.

This study constructed partial least squares regression (PLSR) models based on the same data to evaluate the validity of the models objectively. PLSR is a modeling method that considers both extracting the principal components of the dependent and independent variables and maximizing the correlations between the extracted principal components in the regression process [42,43]. The results of both the MPGA–MIV–BP and the PLSR blood-pressure models are shown in Figure 8 and Table 9. For the MPGA–MIV–BP blood-pressure models, the RMSE of SBP was 3.4043 mmHg, the error (MAD ± STD) was 2.5909 ± 3.4148 mmHg, the RMSE of DBP was 3.2893 mmHg, and the error (MAD ± STD) was 2.6890 ± 3.3117 mmHg. The results met the Association for the Advancement of Medical Instrumentation (AAMI) blood-pressure measurement criteria, which was 5 ± 8 mmHg for MAD ± STD. However, the PLSR blood-pressure models met the STD criteria of AAMI but not the MAD criteria. In addition, the two types of models were evaluated according to the British Hypertension Society (BHS) criteria [44,45], as shown in Table 10. As a result, the SBP and DBP of the MPGA–MIV–BP blood-pressure models were both Grade A, the SBP of the PLSR blood-pressure models was Grade B, while the DBP was only Grade C.

**Figure 9:**
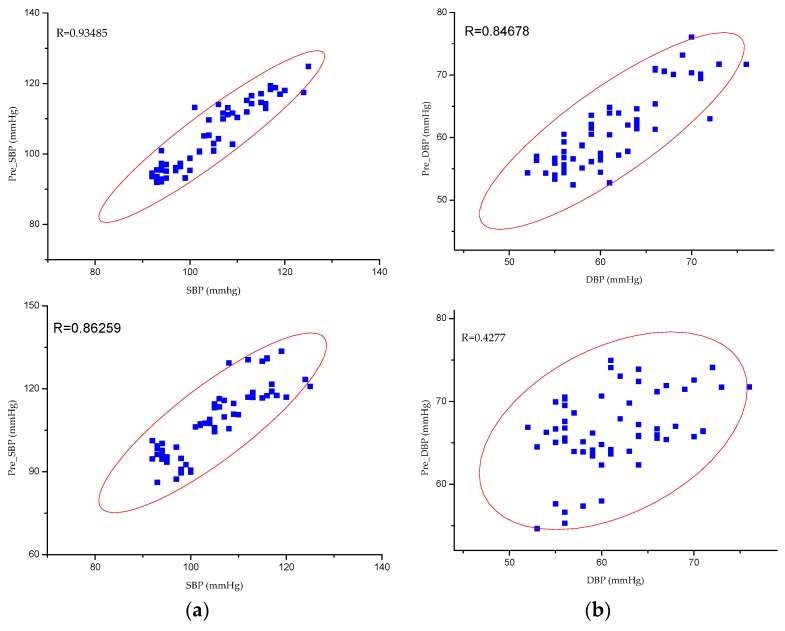
Correlation coefficient of predicted values and actual values. (**a**) SBP models. (**b**) DBP models. For both (**a**,**b**), from top to bottom, MPGA-MIV-BP, PLSR models. Abbreviation: Partial least squares regression (PLSR).

**Figure 10:**
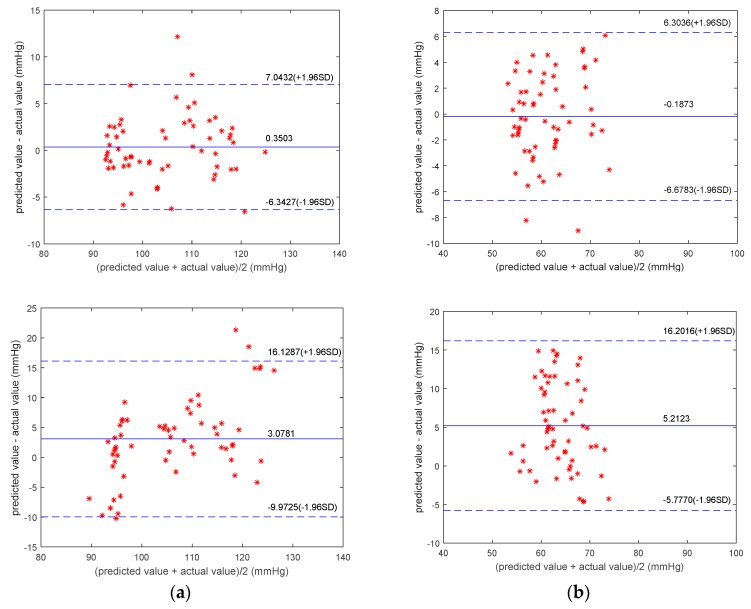
Bland-Altman analysis of predicted values and actual values. (**a**) SBP models. (**b**) DBP models. For both (**a**,**b**), from top to bottom, MPGA-MIV-BP, PLSR models.

This study introduced the Pearson correlation coefficient (denoted as R) analysis [46], as well as Bland–Altman analysis, to better study the correlations between the predicted and measured values for SBP and DBP. For the MPGA–MIV–BP blood-pressure models, the correlation coefficient of SBP and DBP was 0.93485 and 0.84678, respectively, both demonstrating strong correlations between the predicted values and the measured values. In addition, the Bland–Altman analysis of the predictive values and the measured values of SBP and DBP of the MPGA–MIV–BP blood-pressure models showed the mean difference was 0.3503 and −0.1873 (close to 0), indicating no significant differences between the predictions and the measurements. The correlation analysis and the Bland–Altman analysis of the predicted and the measured values of the MPGA–MIV–BP and PLSR blood-pressure models are shown in Figure 9 and Figure 10.

## 4. Discussion

In this study, a set of non-invasive continuous blood-pressure monitoring models based on photoplethysmography and electrocardiography was proposed. Compared with the traditional PWTT-based blood-pressure estimation models, more characteristics (22 waveform characteristics and 2 characteristics of the individuals) were introduced in the parameter selection in model construction, including much more abundant information related to blood pressure. The characteristic parameters were screened so that only the features with large impacts on the model output were included to reduce information redundancy, thus reducing the complexity of the network structure and improving the generalization ability of the network. In view of the disadvantage that the initial connection weights and thresholds that have a great influence on the network cannot be obtained accurately, the MPGA was adopted to determine the initial weights and thresholds of the neural network, which greatly improves the blood-pressure prediction performance of the neural network. Model integration was performed regarding the multiple-trained independent models with various proportional coefficients to reduce overfitting and achieve individualized long-term blood-pressure monitoring. The models in this study were proposed for the prediction of blood pressure over a longer period of time. And the models validation and testing based on the 12 sets of blood-pressure data collected in 20 min showed that the models could be used for long-term blood-pressure monitoring.

By contrast with Tan’s research [37], the optimization method used in this paper is MPGA instead of GA. MPGA is not only used for the optimization of personality parameters, but also for the optimization of neural network. In addition, the personality parameter in this paper is the proportional coefficient of the integrated model, and the correction time is about halved. Finally, the application scenario of the blood-pressure model in this paper is continuous blood-pressure monitoring (20 min) instead of any single point blood-pressure monitoring.

Although the proposed models in this study achieved a certain prediction accuracy, the following issues remained. First, the experiment sample size was small, and all the participants were healthy people. The diversity of experimental data needed to be further expanded. Second, the continuous data collection time of each experiment was limited to 20 min, and the obtained continuous blood-pressure data were only up to 12 data sets. Therefore, the blood-pressure monitoring verification could only be performed for a period of 20 min but not longer, such as half a day, 1 day, or even 1 week. In addition, regarding the characteristics of individuals, this study only considered gender and BMI, while these two characteristics were definitely not sufficient keeping in mind the differences in blood pressure among individuals. Finally, the proportional coefficients need to be calibrated during model integration for different individuals, and the calibration time for a single individual’s blood-pressure models was approximately 1 min.

## 5. Conclusions

The MPGA–MIV–BP monitoring models proposed in this study used MIV to reduce feature redundancy and simplify the model structure. Furthermore, MPGA was used to optimize the initial weights and thresholds of the BP neural network, and determine the proportional coefficients for model integration, effectively reducing overfitting and improving the prediction accuracy of SBP and DBP. The MPGA–MIV–BP monitoring models performed well during long-term (20 min) blood-pressure monitoring (the error of SBP was 2.5909 ± 3.4148 mmHg, and the error of DBP was 2.6890 ± 3.3117 mmHg), providing a direction for the clinical application of non-invasive continuous blood-pressure monitoring equipment.

## Figures and Tables

**Table 1:** Detailed information of the experimental data.

Experimental Data			
Male		Female	
Subject Number	The Number of Data Set	Subject Number	The Number of Data Set
Test1	44	Test14	27
Test2	61	Test15	47
Test3	51	Test16	41
Test4	52	Test17	28
Test5	52	Test18	64
Test6	31	Test19	55
Test7	40	Test20	20
Test8	53	Test21	55
Test9	12	Test22	54
Test10	12	Test23	12
Test11	12	Test24	12
Test12	12	Test25	12
Test13	12	Test26	12
		Test27	12

**Table 2:** The orders of the mean impact value (MIV) values of the input features of the systolic blood-pressure (SBP) model. Abbreviations: Body mass index (BMI); pulse wave transit time (PTT); heart rate (HR).

Oder	Feature	MIV	Cumulative Contribution Ratio
1	BMI	12.5567	0.1600
2	t_downr_	11.5329	0.3069
3	t_bf_	7.4782	0.4022
4	t_fbr_	6.9880	0.4912
5	PTT_c_	4.7147	0.5512
6	t_up_	3.8678	0.6005
7	t_bfr_	3.7754	0.6486
8	t_down_	3.2777	0.6904
9	H_er_	3.1959	0.7311
10	t_aer_	2.6251	0.7645
11	t_upr_	2.6234	0.7979
12	HR	2.5713	0.8307
13	K	2.5514	0.8632
14	PTT_a_	2.4541	0.8945
15	t_ae_	2.4190	0.9253
16	gender	2.0847	0.9518
17	H_fr_	2.0090	0.9774
18	t_fb_	1.7716	1

**Table 3:** The orders of the MIV values of the input features of diastolic blood-pressure (DBP) model.

Oder	Feature	MIV	Cumulative Contribution Ratio
1	t_downr_	10.3509	0.1453
2	H_er_	7.1181	0.2453
3	t_bf_	6.7586	0.3401
4	t_fbr_	6.3810	0.4298
5	BMI	5.5027	0.5070
6	t_bfr_	4.9504	0.5765
7	t_ae_	4.4072	0.6384
8	t_up_	3.5946	0.6889
9	HR	3.2784	0.7349
10	t_aer_	3.1370	0.7790
11	t_fb_	2.4708	0.8136
12	t_down_	2.4611	0.8482
13	t_upr_	2.3780	0.8816
14	PTT_a_	2.0499	0.9104
15	H_gr_	2.0185	0.9387
16	K	1.8294	0.9644
17	H_ar_	1.3742	0.9837
18	PTT_c_	1.1615	1

**Table 4:** The root mean squared error (RMSE) values for SBP of the BP neural network.

Number of Hidden Layer Nodes	RMSE	
	SBP	DBP
5	5.127	3.9945
6	5.1711	3.9554
7	5.0741	3.999
8	4.9535	4.1186
9	4.9923	4.0208
10	4.8541	**3.8598**
11	4.7394	3.861
12	**4.6411**	4.0116
13	4.7493	4.0147
14	4.9888	3.9797
15	4.9229	4.0033

**Table 5:** The RMSE values before and after multiple population genetic algorithm (MPGA) optimization. Abbreviation: Back-propagation (BP).

Netsi/Netdi	RMSE			
	SBP		DBP	
	BP	MPGA_BP	BP	MPGA_BP
1	5.2764	4.0293	4.0228	3.1712
2	5.7259	4.4929	4.2097	3.6848
3	4.5039	3.9589	4.6295	3.7855
4	5.9585	4.2395	4.2171	3.4059
5	4.8591	4.2596	4.9986	3.8372

**Table 6:** The proportional coefficients of SBP prediction models of different individuals.

	$a_{1}$	$a_{2}$	$a_{3}$	$a_{4}$	$a_{5}$	RMSE
Test9	0.1812	0.32817	0.5383	–0.27353	0.27329	1.6911
Test10	–0.31502	0.75836	–0.043286	–0.20529	0.79999	3.0771
Test11	0.56489	–0.15901	–0.45616	0.39878	0.63647	2.0115
Test12	–0.061829	0.18831	0.79962	0.0011497	0.18906	2.7134
Test13	0.20237	–0.46849	0.062964	0.51146	0.52279	4.5162
Test23	–0.13989	0.78121	–0.13627	0.64605	–0.4069	1.6057
Test24	0.18856	0.34317	–0.31996	0.26478	0.58522	1.423
Test25	–0.5	–0.28147	0.24398	0.79993	0.62657	1.855
Test26	0.010734	0.79722	–0.057566	0.63801	–0.5	2.5736
Test27	0.10024	0.3309	0.61282	0.20206	–0.23555	0.92414

**Table 7:** The proportional coefficients of DBP prediction models of different individuals.

	$b_{1}$	$b_{2}$	$b_{3}$	$b_{4}$	$b_{5}$	RMSE
Test9	–0.28713	0.085775	0.59742	0.1077	0.1077	1.0499
Test10	0.21619	–0.46177	0.6959	0.14974	0.38735	3.2466
Test11	0.16096	0.13109	–0.039645	0.79989	–0.28671	1.8712
Test12	0.4358	0.45117	0.32725	–0.33487	0.13734	0.57529
Test13	0.40708	0.30307	–0.14565	0.41061	0.011025	3.6798
Test23	0.63959	0.09538	0.47616	0.086609	–0.49953	2.4961
Test24	0.10676	0.66158	–0.21585	0.36542	–0.089198	0.83838
Test25	0.70866	0.25386	–0.2732	0.4427	–0.18105	0.96062
Test26	0.31308	0.16157	0.77251	–0.088219	–0.029835	0.53645
Test27	0.59681	0.30045	–0.12228	0.21974	–0.016416	3.1992

**Table 8:** The model prediction results. Abbreviations: Root mean squared error (RMSE); mean absolute deviation (MAD); standard deviation (STD).

	SBP (mmHg)			DBP (mmHg)		
	RMSE	MAD	STD	RMSE	MAD	STD
Test9	3.3667	2.7483	3.6798	3.5002	3.1502	3.1158
Test10	1.9918	1.6463	2.0956	4.2858	4.0309	4.0473
Test11	6.03	4.7696	4.0416	2.3613	1.726	2.5566
Test12	2.6706	2.3583	2.603	4.9515	4.5651	2.1008
Test13	2.7154	2.2694	2.9716	4.346	3.4716	4.7155
Test23	1.7656	1.6088	1.5235	1.4618	1.3235	1.5291
Test24	2.1268	1.593	1.7522	2.5727	2.3415	2.294
Test25	2.7512	2.117	1.9318	2.3513	1.8745	1.7989
Test26	4.7518	4.1512	5.1785	2.7733	2.2527	2.5884
Test27	3.4544	2.6524	2.7124	2.5732	2.1541	1.8145

**Table 9:** Comparison of models.

	SBP (mmHg)			DBP (mmHg)		
	RMSE	MAD	STD	RMSE	MAD	STD
MPGA-MIV-BP	3.4043	2.5909	3.4148	3.2893	2.6890	3.3117
PLSR	7.2850	5.5768	6.6585	7.6210	6.1186	5.6068

**Table 10:** Grading of: British Hypertension Society (BHS) system and the grades of different models.

Model		Cumulative Probability Distribution (%)			Grade
		≤5 mmHg	≤10 mmHg	≤15 mmHg	
BHS	SBP/DBP	60	85	95	A
		50	75	90	B
		40	65	85	C
MPGA-MIV-BP	SBP	86.667	98.3333	100	A
	DBP	90	100	100	A
PLSR	SBP	55	86.667	95	B
	DBP	53.3333	73.333	100	C
